# Radiomics Assessment of the Tumor Immune Microenvironment to Predict Outcomes in Breast Cancer

**DOI:** 10.3389/fimmu.2021.773581

**Published:** 2022-01-03

**Authors:** Xiaorui Han, Wuteng Cao, Lei Wu, Changhong Liang

**Affiliations:** ^1^ School of Medicine, South China University of Technology, Guangzhou, China; ^2^ Department of Radiology, Guangdong Provincial People’s Hospital, Guangdong Academy of Medical Sciences, Guangzhou, China; ^3^ Department of Radiology, The Sixth Affiliated Hospital, Sun Yat-Sen University, Guangzhou, China; ^4^ Guangdong Provincial Key Laboratory of Artificial Intelligence in Medical Image Analysis and Application, Guangzhou, China

**Keywords:** radiomics, breast cancer, DCE-MRI, immune microenvironment, immunoscore

## Abstract

**Background:**

The immune microenvironment of tumors provides information on prognosis and prediction. A prior validation of the immunoscore for breast cancer (IS_BC_) was made on the basis of a systematic assessment of immune landscapes extrapolated from a large number of neoplastic transcripts. Our goal was to develop a non-invasive radiomics-based IS_BC_ predictive factor.

**Methods:**

Immunocell fractions of 22 different categories were evaluated using CIBERSORT on the basis of a large, open breast cancer cohort derived from comprehensive information on gene expression. The IS_BC_ was constructed using the LASSO Cox regression model derived from the Immunocell type scores, with 479 quantified features in the intratumoral and peritumoral regions as observed from DCE-MRI. A radiomics signature [radiomics ImmunoScore (RIS)] was developed for the prediction of IS_BC_ using a random forest machine-learning algorithm, and we further evaluated its relationship with prognosis.

**Results:**

An IS_BC_ consisting of seven different immune cells was established through the use of a LASSO model. Multivariate analyses showed that the IS_BC_ was an independent risk factor in prognosis (HR=2.42, with a 95% CI of 1.49–3.93; P<0.01). A radiomic signature of 21 features of the IS_BC_ was then exploited and validated (the areas under the curve [AUC] were 0.899 and 0.815). We uncovered statistical associations between the RIS signature with recurrence-free and overall survival rates (both P<0.05).

**Conclusions:**

The RIS is a valuable instrument with which to assess the immunoscore, and offers important implications for the prognosis of breast cancer.

## Introduction

The tumor immune microenvironment (TIME) displays key actions in tumor development, metastasis, and the response to therapy (1, 2). Many researchers have confirmed the prognosis and potentially predictive importance of the infiltration of immune cells into tumors (3–6). Currently, the assessment of immune infiltration, such as immunoscore testing, usually requires post-surgically acquired tissue samples. Due to the dynamic character of the immune reaction (7), assessment of TIME through non-invasive methods would be helpful and allow for a longitudinal evaluation regarding the immune infiltrate across the entire therapeutic course.

Radiography entails a wealth of knowledge comprising tumor phenotypes (8) that are not only controlled by the inherent biology of tumor cells but also regulated by the tumor microenvironment (TME). Analysis of radiologic images by quantified radiomics methods can reveal associations between particular images with molecular phenotypes (9). And some investigators have already begun exploring the relationships between imaging features and tumor-infiltrating lymphocytes (10–13).

Breast cancer continues to be the commonest cancer worldwide, and the second leading cause of cancer-related deaths (14). Clinicopathologic risk factors cannot currently be used to precisely predict outcome, and more accurate risk stratification is thus required for the appropriate timing of surgery and the implementation of chemotherapeutic regimens (15).

We are currently developing an ImmunoScore for breast cancer (IS_BC_) based upon patient RNA sequencing information, and then validating the IS_BC_ as a reliable and independent prognostic predictor. We thereby assume that radiomics will permit a non-invasive assessment of TIME. A two-fold aim of our study was (a) to establish a radiomic signature of the IS_BC_, and (b) to evaluate the capability of the IS_BC_ in predicting survival.

## Patients and Methods

### Collection of Data Cohorts

The image datasets were gathered from The Cancer Imaging Archive (TCIA) open-access dataset, and the respective gene-expression profiles were acquired through The Cancer Genome Atlas (TCGA). Motivated by prior studies (16–18) that indicated that TIME is correlated with the prediction of breast cancer, we created and verified the association of imaging phenotypes with TIME by using three datasets. There was zero patient overlap across the three datasets, and descriptive and clinical statistics of all three cohorts are shown below in **Table 1**.

**Table 1:** Characteristics of patients in the TCGA, radiogenomic, and validation cohorts.

Variables	TCGA cohort		Radiogenomic Cohort		Validation cohort	
	n = 335		n = 120		n = 155	
	N	%	N	%	N	%
Age (years)						
18-60	194	57.9	80	66.7	137	88.4
>60	141	42.1	40	33.3	18	11.6
Laterality						
Left	165	49.3	61	50.8	77	49.7
Right	170	50.7	59	49.2	78	50.3
Race						
White	216	64.5	97	80.8	143	92.3
Black or African American	70	20.9	22	18.3	4	2.6
Asian	21	6.3	1	0.8	8	5.2
Other	28	8.4	/	/	/	/
Status						
Alive	286	85.4	117	97.5	132	85.2
Dead	49	14.6	3	0.5	21	13.5
Lost	0	0	0	0	2	1.3
OS(years)						
≤1	50	14.9	6	5.0	5	3.2
>1 ≤3	151	45.1	50	41.7	21	13.6
>3 ≤5	58	17.3	31	25.8	102	65.8
>5years	70	20.9	30	25.0	27	17.4
Unknown	6	1.8	3	2.5	0	0
Depth of invasion						
pT1	70	20.9	48	40.0	/	/
pT2	220	65.7	66	55.0	/	/
pT3	33	9.9	6	5.0	/	/
pT4	12	3.6	0	0	/	/
Lymph node metastasis						
pN0	166	49.6	63	52.5	/	/
pN1	107	31.9	41	34.2	/	/
pN2	36	10.7	9	7.5	/	/
pN3	21	6.3	6	5.0	/	/
pNx	5	1.5	1	0.8	/	/
Metastasis						
pM0	277	82.7	94	78.3	/	/
pM1	6	1.8	0	0	/	/
pMx	52	15.5	26	21.7	/	/
Stage						
I	49	14.6	28	23.3	/	/
II	206	61.5	76	63.4	/	/
III	69	20.6	16	13.3	/	/
IV	6	1.8	0	0	/	/
Unknown	5	1.5	0	0	/	/
Estrogen receptor status						
Positive	206	61.5	99	82.5	88	56.8
Negative	116	34.6	21	17.5	65	41.9
Indeterminate	0	0	0	0	0	0
Unknown	13	3.9	0	0	2	1.3
Progesterone receptor status						
Positive	173	51.6	88	73.3	74	47.7
Negative	148	44.2	32	26.7	79	51.0
Indeterminate	1	0.3	0	0	0	0
Unknown	13	3.9	0	0	2	1.3
Human epidermal growth factor receptor 2 status						
Positive	60	17.9	13	10.8	47	30.3
Negative	184	54.9	62	51.7	105	67.7
Indeterminate	46	13.7	26	21.7	0	0
Unknown	45	13.4	19	15.8	3	2.0
Neoadjuvant chemotherapy						
YES	316	94.3	1	0.8	153	98.7
NO	0	0	119	99.2	2	1.3
Unknown	19	5.7	0	0	0	0

The first dataset, called the TCGA cohort, consists of the data from 335 individual cases gathered from the TCGA database, together with RNA sequencing data from cancer specimens as well as Recurrence-free survival (RFS) and overall survival (OS); however, there are no data with respect to imaging. This cohort was then randomly partitioned into a training set (~70%) and a validation set (~30%).

The second dataset, referred to as the Radiogenomic Cohort, originally consisted of 137 cases of patients who had usable DCE-MRI images of TCGA-BRCA, along with the appropriate gene expression information in the TCGA dataset. One patient with no usable gene expression data, seven without usable clinical details, and nine whose imaging was not complete were deleted from the study. The finalized dataset consisted of 120 patients, and these were allocated to training and validation sets in a ratio of 8:2.

We enrolled a validation cohort consisting of 222 breast cancer cases (from the I-SPY 1 TRIAL in the TCIA database), together with usable DCE-MRI and appropriate RFS and OS information. We eliminated 26 cases of patients who had incomplete image sequences, 10 patients without measurable neoplasms, and 31 manifesting poor image quality. The resulting dataset thus encompassed a panel of 155 breast cancer patients.

Data in the TCGA and TCIA databases are open access, and our study adhered to the data- accessibility policies and release guidance with respect to both databases, and therefore did not require approval from the local ethics committee.

### Outline of the Framework

**Figure 1:**
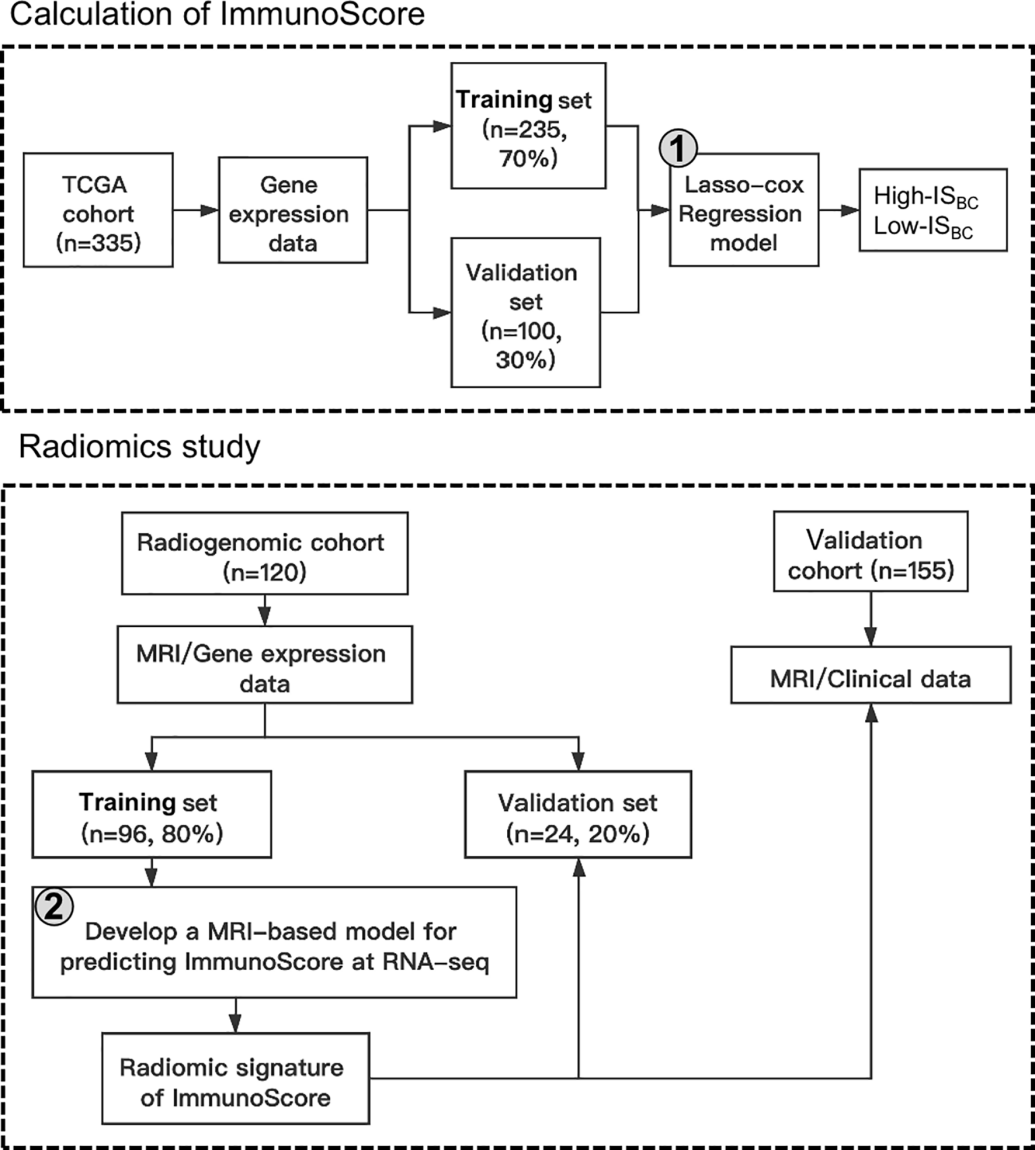
Design of the study in which a breast cancer ImmunoScore was developed and used to validate the radiomic signature.

As **Figure 1** illustrates, the framework of our research consisted of two blocks: (i) calculation of an immunoscore based on RNA sequencing information, and (ii) development of a radiomic feature (radiomics ImmunoScore [RIS]) for non-invasive assessment of the cancer immunoscore, and evaluation of the capability of the RIS to predict survival.

### Calculation of Immunoscore

We used the CIBERSORT algorithm to calculate the patient’s immunoscore as previously described, the procedure of which can be found in **Supplementary Material** (19–21). This method is designed to work effectively and is already proven on gene expression spectra as measured with microarrays. In the present study, scores of 22 immune cell categories were assessed using CIBERSORT on a series of breast cancer gene expression profiles according to clinical notes. The imputed scores of immune cell groups generated from CIBERSORT were deemed to be exact under a threshold of P < 0.050. A patient was therefore deemed qualified to undergo further analysis only if CIBERSORT P <0.050. The immunoscore was then created using a Least Absolute Shrinkage and Selection Operator(LASSO) Cox regression analysis.

### Image Collection and Tumor Segmentation

MR images were downloaded from the TCIA database (for MR acquisition parameters, see **Supplementary Material**). Two diagnostic imaging physicians (XR and WT, with 5 and 9 years of diagnostic breast MRI experience, respectively) manually displayed the primary neoplasm on MRI images with ITK-SNAP software. In order to obtain infiltrative-margin data, a ring was formed around the primary neoplasm, with the tumor margin automatically expanded outward by 2 mm and the tumor margin contracted inward by 1 mm to form a ring of 3 mm thickness. The macrovasculature, neighboring organs, and air spaces were excluded (**Figure S1**).

### Feature Extraction

Radiomics features were defined based on the PyRadiomics Python package, version 1.2.0 (22), and analyzed using the recommended set of defaults. We extracted 479 quantified features of a patient’s MR images separately in each of the regions of interest, i.e., peritumoral and intratumoral regions, and evaluated them by calculating the AUC (**Figure S2**). The extracted features included 90 first-order features, 14 shaped features, as well as 375 second- and higher-order textural features. The four textured features we studied were based on wavelet decomposition of the grayscale co-occurrence matrix, grayscale run-length matrix, grayscale size-region matrix—as well as the neighborhood grayscale-difference matrix. Image features with various spatial scales were derived by rotating the filter parameters (2.0, 2.5, 3.0, and 3.5) between 2.0 and 3.5 using a Gaussian spatial bandpass filter (∇2G).

### Feature Selection

Inter-observer and intra-observer consistency was performed by analyzing all radiomics features extracted based on intra and interclass correlation coefficients (ICCs). Thirty patients were randomly selected, features of which were extracted by radiologists XR and WT. The same steps were also repeated for two weeks by radiologist XR. ICC > 0.8 suggested good agreement.

Recursive feature elimination was employed for selecting the most helpful prediction features among the primary dataset.

### Construction of a Radiomics Immunoscore

Using the training set of Radiogenomic Cohort, we built a random forest model to predict the RNA-Seq-based immunoscore [radiomics ImmunoScore (RIS)], which was selected as the optimal model by a five-fold cross-validation. This model was executed on the validation set, with an optimal threshold for the RIS using the Youden index, which optimized the total sensitivity and specificity.

### Statistical Analyses

Comparisons between the two groups were made by Student’s t-test for continuous variables and either Chi-squared or Fisher exact-probability tests for categorical variables. Kaplan-Meier method-based survival curves were produced and compared using log-rank tests. We used Cox proportional risk models for univariate and multivariate analyses. LASSO-Cox regression analysis was performed for constructing the immunoscore for breast cancer. A random forest classifier model was used to classify the immunoscore. Model accuracy was evaluated with the AUC. Inter-observer and intra-observer consistency was performed by ICCs. Error detection rates were computed to obtain corrected P-values in multiple comparisons. We employed R 3.4.0 and SPSS 22.0 for statistics, and bilateral P-values <0.05 were regarded as significant.

## Results

### Demographic Characteristics

The selected protocols for the TCGA cohort patients are presented in **Figure S3**. Following application of the data-screening criterion, overall survival data from 335 clinically annotated breast cancer specimens were accessible for additional analyses. Details of the patient demographics are shown in **Table 1**.

Detailed clinicopathologic features for individuals in the Radiogenomic Cohort (n=120) and Validation Cohort (n=155) are presented in **Table 1**. The median age (interquartile range) of the 275 patients enrolled in this study was 51.0 (44.0–59.0) years.

### Estimation of the Immunoscore

**Figure 2:**
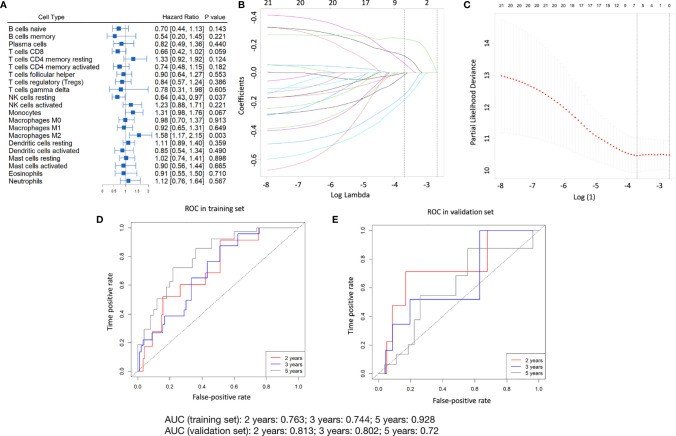
Construction of the immunoscore model. **(A)** The forest plot shows the relationship of different subpopulations of immune cells to OS in the training set. **(B)** Distribution of LASSO factors for 21 immune cell fractions. The dashed curve represents values selected *via* 10-fold crossover validation. **(C)** Crossover validation of a 10-fold choice of adjustment parameters from the LASSO model. The bias likelihood deviation was expressed in log(λ) whenever λ was the adjustment parameter. Values of the bias-likelihood deviation are displayed, and the error bands indicate S.E. of the mean according to the minimal criterion and the 1-S.E. criterion; vertical dashed lines were plotted at the optimal point. Numbers at the top denote numbers for cell categories implicated in the LASSO model for **(B, C)** The prognostic accuracy of the immunoscore as a continuous variable as assessed by ROC analysis in the training set **(D)** and validation set **(E)**.

We employed the survminer software package on the TCGA cohort training set (235 patients) for generating the best cutoff values per immune cell fraction. A forest plot showing the correlation between every immune cell sub-population and overall survival is shown in **Figure 2A**. The immunoscore was modeled on the training set using LASSO-Cox regression analyses (**Figures 2B, C**) (see **Supplementary Material** for the formula used to calculate the immunoscore). Time-dependent ROC analyses were performed at the 2-, 3-, and 5-year time-points to study the accuracy of the prognosis of the immunoscore as a continuous variable in the training set (**Figure 2D**), and the corresponding AUC values and calibration curve are shown in **Figures S4A, B**. The cut-off (-0.115) derived by the survminer package was then utilized to classify the patients in the training set into high and low immunoscore groups. The results of the five-year survival analysis of different immunoscore groups, different age groups, and different pathologic stages are shown in **Figure S5**. The results of our multivariate Cox regression analysis regarding the correlation between immunoscore and overall survival are depicted in **Table S1**.

### Validation of Immunoscore Prediction of Survival in the TCGA Cohort-Validation Set

The identical equation was used in the validation data set of the TCGA cohort in order to verify a similar prognostic value for the constructed immunoscore model across populations. In the validation set, the prognostic precision regarding the immunoscore used as a continuous variable was also evaluated *via* time-dependent ROC analyses (**Figure 2E**).

### Construction and Validation of Radiomics Immunoscore

Both intra- and inter-observer ICCs were greater than 0.8, indicating good reproducibility of feature extraction.

**Figure 3:**
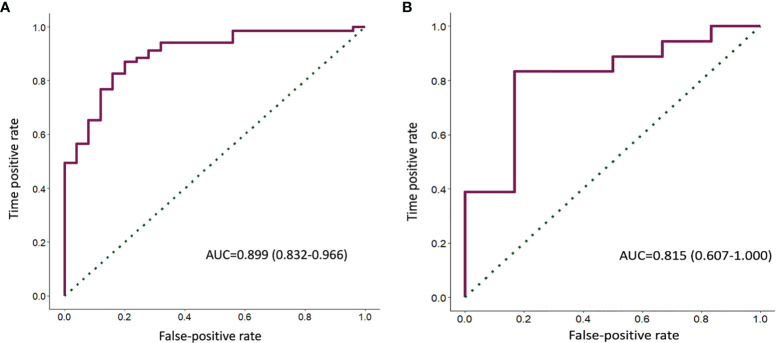
ROC curves of the RIS predicted the ISBC in both the training **(A)** and validation sets **(B)**.

A random forest was used to construct the classification for the IS_BC_ in the training set of the Radiogenomic Cohort. Select the top 10 features in terms of feature importance and plot the feature relative importance histogram (**Figure S6**). The resulting radiomics signature (RIS) consisted of 21 predictors with six marginal features and 15 intratumoral features (**Table S2**). In the training set, the capability of the RIS to classify high IS_BC_ and low IS_BC_ showed an AUC of 0.899 (95% confidence interval [CI], 0.832–0.966) (**Figure 3A**). The radiomics signature revealed a similar accuracy in predicting the IS_BC_ in the validation set with an AUC of 0.815 (95% CI, 0.607–1.000) (**Figure 3B**). The RIS, however, exhibited a higher AUC value than any single radiomics feature (**Figure S7**). In addition, the AUC of the RIS was compared with the volume and diameter of the core and infiltration zones of the validation set (**Figure S8**). The optimal cut-off for the RIS in the training set was 0.686 as defined by the ROC curve (**Figure 3A**). Therefore, patients were classified into a low-RIS group when their RIS was <0.686, and a high-RIS group when their RIS was ≥0.686. The association between the RIS and clinicopathologic characteristics is shown in **Table S3**.

### Prognostic Value of Radiomics ImmunoScore

**Figure 4:**
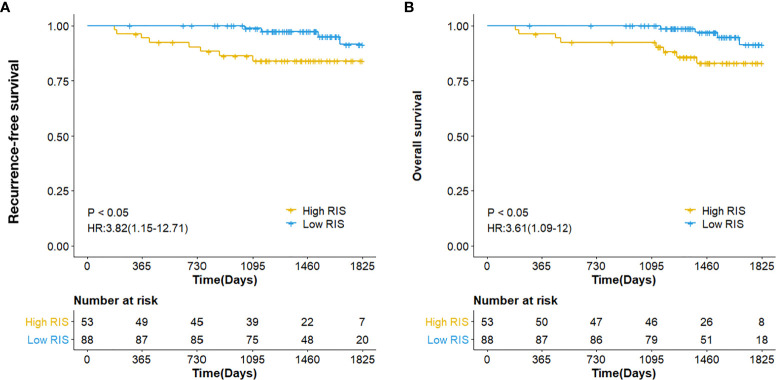
Kaplan-Meier analysis for RFS **(A)** as well as OS **(B)**, depending upon the RIS dichotomous signature of the breast cancer patients.

The prognostic value of the RIS was then evaluated in our validation cohort. The five-year RFS and OS in the low-RIS group were 91.49% and 91.32%, respectively, and these survival indices in the high-RIS group were 84.09% and 82.94%, respectively (**Figure 4**), indicating that the prognoses for patients who were stratified on the basis of the RIS were significantly different.

We conducted multivariate Cox regression analyses and adjusted for clinicopathologic variables. In the validation cohort, the RIS continued to be a strong and independent prognostic predictor of RFS and OS (**Table 2**). Finally, we noted that the combined model-integrating radiomics and clinicopathologic features enhanced the prognostic accuracies of the validation cohort (**Figure S9** and **Tables S4, S5**).

**Table 2:** Cox regression analysis of multivariate for RFS and OS of breast cancer patients.

Variables	RFS		OS	
validation cohort	95%CI	p	95%CI	p
RIS (high vs. low)	0.079-0.870	0.029	0.083-0.920	0.036
Estrogen receptor status(positive vs. negative)	0.564-1.088	0.145	0.542-1.046	0.009
Progesterone receptor status(positive vs. negative)	0.607-1.162	0.293	0.610-1.168	0.306
Human epidermal growth factor receptor2 status(positive vs. negative)	0.572-3.835	0.419	0.547-3.655	0.474
Laterality(left vs. right)	0.379-2.407	0.922	0.389- 2.470	0.967
Age(≥60 vs. <60)	0.429-5.126	0.533	0.446-5.322	0.495

## Discussion

The immune microenvironment of tumors is a critical determining factor in the therapeutic response and results in numerous types of cancers (23), and high-level immune infiltration has been shown to be related to positive clinic results in BC (24). In the present study, we calculated an immunoscore using CIBERSORT, an algorithm that can accommodate high numbers of cancer samples that have already been analyzed by RNA sequencing so as to estimate proportions of immune cells. This algorithm avoids the complex process of immunostaining and offers a substitute for flow or mass cytometry-based approaches. Importantly, archives of RNA and cellular samples are equally accessible to CIBERSORT (25). It has also been demonstrated that CIBERSORT is effective in identifying particular immune subgroups, representing a major advance compared to other methods that reflect more restricted capabilities (26–28). In the present study, an immunoscore model consisting of seven immune cells was constructed and evaluated by applying LASSO regression (29, 30), and the predictive value was validated in both the training as well as the validation set. We showed a significant segregation between OS curves of high and low immunoscore subjects, which is in line with previous studies (31). Furthermore, the ability of the ImmunoScore in predicting patient group survival, similar to TNM staging, suggests this model may be useful for prognostic purposes and could possibly supplement the current TNM staging approach.

Our assessment of the immune microenvironment in the tumors was determined for histologic samples, was only available on a single postoperative basis, and was restricted by the inherent heterogeneity of the biopsied tissue. By comparison, the rare benefit of radiographic images is that they are available non-invasively and can be obtained continuously prior to and across the course of therapy. Radiomic analyses can uncover microscopic tumor profiles that mirror the makeup of tumor-invading immune cells. In our study, we found that RIS can be used to predict breast cancer immunoscore and confirmed in the validation set, suggesting that radiomics is feasible for predicting breast cancer immunoscore. While image-based assessments will likely neither supplant nor substitute for the current gold standard of tissue-based assessment, we posit that our radiographic methodology would be helpful in promoting long-term evaluation and *in vivo* surveillance of the TME. In fact, some researchers have surveyed the relationship between radiographic features and tumor-invading lymphocytes (10–12), and the ability of radiomic features to predict prognosis (12). Ferté et al. correlated both on-tumor and peri-tumor radiomic features with CD8 expression at the central tumor area and suggested that imaging features might help assess the CD8 cell population and also forecast the clinical response for those patients receiving antibody therapy (11). In another study, Tang et al. related intra-tumor radiomics to critical immunologic profiles (32). In addition, RFS as well as OS curves were significantly separated based on the RIS dichotomous characteristics of breast cancer patients and patients with low RIS had a better prognosis, which is consistent with the literature (33).

The radiomic signature presented in this study was defined using preprocessed MRI images that reflected the potential biologic (principally immune-related) features of the TME unrelated to therapy (33). Therefore, the radiomic signature developed during diagnostic imaging might also apply to clinical settings that encompass multiple treatment regimens. Further efforts will be required to evaluate the radiomic signature within these specified settings.

A major advantage to our work was that when we deduced a radiomic signature, we not only executed an analysis of the imaging features within the tumor alone but also clearly identified the structure of the circumferential ring around the peritumoral area. The reason for this was that the peritumor environment secretes large amounts of growth factors and cytokines, which can induce oxygen deprivation and angiogenesis, playing important functions in tumor development, progression or metastasis. Integrating tumor and peritumor data can more comprehensively portray the aggressive and metastatic characteristics of tumors. Thus, extraction and fusion of tumor and peritumor features can be improved as the predictive properties of radiomics models (34). Similar radiomic methods are already employed to exploit radiomic signatures for the purpose of forecasting chemotherapeutic reactions in gastric cancer (33). The utilization of sophisticated deep-learning technologies also contributes to the automated identification of new imaging phenotypes in forecasting results (35).

Although we uncovered several significant elements, there were still some limitations to our study that need to be resolved. First, the size of our patient cohort remained comparatively small, as there were only a restricted number of usable and accessible RNA sequencing information and breast MR images from the TCGA as well as the TCIA databases. The predictive accuracy of imaging signatures in predicting the IS_BC_ remains to be validated by additional extrinsic research in this area. Next, data from the DCE-MRI were obtained in a multisite cohort that possessed different imaging characteristics and provided a variety of images. Finally, since all subjects in our study were chosen retrospectively, prospective randomized trials are required in the future to validate our findings.

Overall, we established a radiomic signature that enabled us to non-invasively assess TIME, particularly the immunoscore. Studying radiomic features to forecast and detect immunotherapeutic reactions may therefore constitute an attractive area of focus when considering the dynamic quality of the immune reaction.

## Data Availability Statement

The original contributions presented in the study are included in the article/**Supplementary Material**. Further inquiries can be directed to the corresponding author.

## Author Contributions

XH carried out the study design. XH and WC conducted the experiments. LW helped to analyze the data. CL provided experimental assistance. XH wrote the manuscript. CL supervised the overall project. CL revised the manuscript. All authors contributed to the article and approved the submitted version.

## Funding

This work was supported by the Key R&D Program of Guangdong Province, China (grant number: 2021B0101420006); the National Key R&D Program of China (grant number: 2017YFC1309100); National Natural Science Foundation of China (grant number: 82071892); High-level Hospital Construction Project (grant number: DFJH201805); Project Funded by China Postdoctoral Science Foundation (grant number: 2020M682643); the National Science Foundation for Young Scientists of China(grant number: 82102019).

## Conflict of Interest

The authors declare that the research was conducted in the absence of any commercial or financial relationships that could be construed as a potential conflict of interest.

## Publisher’s Note

All claims expressed in this article are solely those of the authors and do not necessarily represent those of their affiliated organizations, or those of the publisher, the editors and the reviewers. Any product that may be evaluated in this article, or claim that may be made by its manufacturer, is not guaranteed or endorsed by the publisher.
