# Supplementary material for: Radiomics Assessment of the Tumor Immune Microenvironment to Predict Outcomes in Breast Cancer
Source: Front Immunol. 2022 Jan 3;12:773581. doi: 10.3389/fimmu.2021.773581 (PMC8761791; doi:10.3389/fimmu.2021.773581)
Supplement: Supplementary file 1 [file DataSheet_1.doc]

Supplementary Material

# Supplementary Data

## Patients

Our study design as a whole is presented on Figure 1. we reviewed a total of 610 breast cancer patients from the TCIA and TCGA public databases. All patients had histologically proven breast cancer and had full clinicopathologic characteristics information as well as follow up data. Also, it was excluded patients whose neoplastic lesions were not identifiable on MR images.

Since patients from the validation cohort did not have gene expression information, it was used for validating the prognostic worth of RIS, while not for evaluating the predictability of RIS by ImmunoScore.

## Estimate the fraction for immune cell types

In order to quantitate the percentage of immune cells within breast cancer specimens, we have employed the CIBERSORT approach together with the LM22 gene signature[1];which comprises 547 genes that can highly sensitively and specifically discriminate between 22 human haemopoietic cell types, which include the B-cell, T-cell, natural killer cell, macrophage, dendritic cell and myeloid sub-populations. CIBERSORT using Montecarlo drawing for generating P-values for the inverse convolution for individual samples, which provides the metric for confident results. [1, 2]. The percentage of immune cells was individually predicted to every series of gene expression. The sum of the estimated values for all immune cell type scores was equal to 1 on each specimen.

Based on the lasso regression model, we derived the following formula for calculating IS_BC_ per patient: IS_BC_= (0.26624×fraction level of T cells CD4 memory resting)-(0.41478×fraction level of NK cells resting)-(0.21822×fraction level of B cells naive)+(0.18178×fraction level of Monocytes)-(0.08543×fraction level of T cells CD4 memory activated)+(0.25964×fraction level of Macrophages M2)-(0.34107×fraction level of T cells CD8). Within this equation, the level of the immune cell fraction was specified either as 0 or 1; 0 was given when a category's fraction was lower than the respective cutoff value, or 1 otherwise. With MR images and gene expression data available in the training cohort, it is possible to estimate the number of immune cells and perform radiomics analyses on the correspondent images of tissue samples from tumor biopsies.

## MR image collection and processing

### Imaging protocol

In the radiogenomic cohort, DCE-MRI sequences were collected in the TCGA-BRCA dataset. The scan protocol includes one preenhanced as well as three to five postenhanced images that were acquired with T1-weighted, 3D degenerate gradient echo sequences using gadolinium based contrasts. Typically, the intraplanar solution is 0.53 to 0.85 mm, with a layer spacing of 2 to 3 mm and a 10° flipping angle.

In the validation cohort, MR images were acquired in a 1.5T scanner with a breast coil. The enhanced T1-weighted sequences were obtained at the sagittal position. The parameters of the fat-suppressed gradient echo series were. TR/TE=20/4.5 (ms), FOV 160-180 mm, flipping angle ≤45°, minimal matrix 256 × 192, number of layers 64, layer thickness ≤2.5 mm, intraplanar space solution ≤1 mm. Precontrast sequences were first collected, then 2.5 and 7.5 min post-contrast agent injection were collected for early and delayed phase sequences.

### Feature extraction and selection

Considering an outside estimator assigning the weight to a feature ( for example, coefficient for a linear model), recursive feature elimination (RFE) targets the selection of the feature through recursive consideration of smaller to smaller feature collections. Initially, estimators are training over an initially feature set, where each feature's importance has been derived by any particular property (e.g., coef_, feature_importances_) or callability. After that, lowest importance features are trimmed out from present feature set. This process repeats recursively over the trimmed set till finally reaching the required feature number for selection.[3, 4]. RFECV executes RFE during the cross validation loops for finding the optimum feature count.

A five-fold cross-validation has been used in the selection of features and its evaluation by AUC was calculated, and the corresponding plots were obtained by recording the AUC under different number of features in the feature selection process (**Figure. S7**). 21 features were finally selected (**Table S2**).

### Construction of Radiomics Signature using random forest Model

The Radiogenomic Cohort was divided into training and test sets according to 8:2. In the training set, the optimal model was selected by 5-fold cross-validationa, followed by testing on the test set.

Model diagnostic capability was evaluated using the AUC, which measured the the model's ability to discriminate IS_BC_ as 0 for low IS_BC_ or 1 for high IS_BC_. The optimum cut-off for RIS was identified in the training set by using the Youden index[5].

The importance of features on the basis of impurities. The higher it is, the more importance a feature has. A feature's importance was calculated based on the standardized (normalized) overall decrease caused from that feature. Also called Gini importance.[6] We selected the top 10 features in terms of feature importance and plotted the feature relative importance histogram (**Figure S6**).

## Integrated Nomogram

We developed an integrated nomogram incorporating radiomics and clinicopathology features. his nomogram included raidomic signature and clinicopathological risk factors (ER receptor status + PR receptor status). Harrell's concordance index (C-index) has been calculated in order to quantitate the discriminatory properties[7] . For comparing predicted and real survivals, calibration curves were plotted.

## References

1. Newman, A.M., et al., *Robust enumeration of cell subsets from tissue expression profiles.* Nat Methods, 2015. **12**(5): p. 453-7.

2. Ali, H.R., et al., *Patterns of Immune Infiltration in Breast Cancer and Their Clinical Implications: A Gene-Expression-Based Retrospective Study.* PLoS Med, 2016. **13**(12): p. e1002194.

3. Jiang, Y., et al., *Noninvasive imaging evaluation of tumor immune microenvironment to predict outcomes in gastric cancer.* Ann Oncol, 2020. **31**(6): p. 760-768.

4. Trebeschi, S., et al., *Predicting response to cancer immunotherapy using noninvasive radiomic biomarkers.* Ann Oncol, 2019. **30**(6): p. 998-1004.

5. Reibnegger, G. and W. Schrabmair, *Optimum binary cut-off threshold of a diagnostic test: comparison of different methods using Monte Carlo technique.* BMC Med Inform Decis Mak, 2014. **14**: p. 99.

6. Kniep, H.C., et al., *Radiomics of Brain MRI: Utility in Prediction of Metastatic Tumor Type.* Radiology, 2019. **290**(2): p. 479-487.

7. Mayr, A., B. Hofner, and M. Schmid, *Boosting the discriminatory power of sparse survival models via optimization of the concordance index and stability selection.* BMC Bioinformatics, 2016. **17**: p. 288.

# Supplementary Figures and Tables

## Supplementary Figures

**
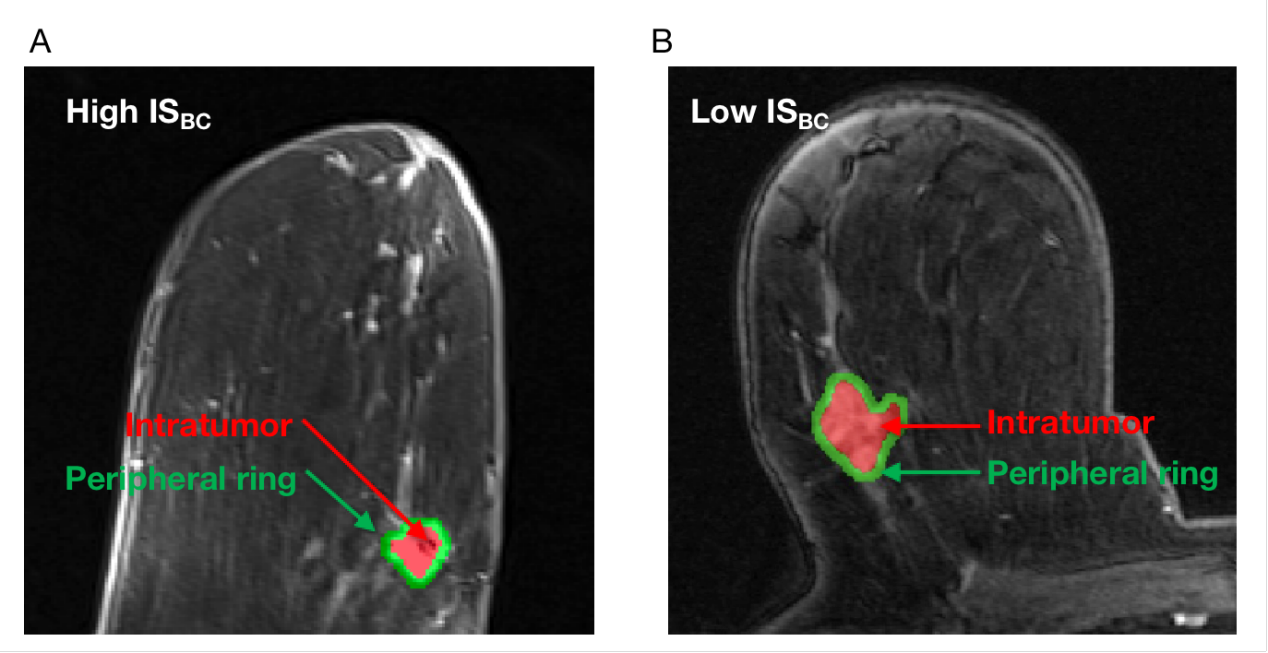
**

**Supplemental figure 1.** Representative MRI images in the high and low immunoscore groups. **(A)** Case one, Stage IIA, T1cN1aM0, High IS_BC_. **(B)** Case two, Stage IIB, T3N0Mx, Low IS_BC_

**
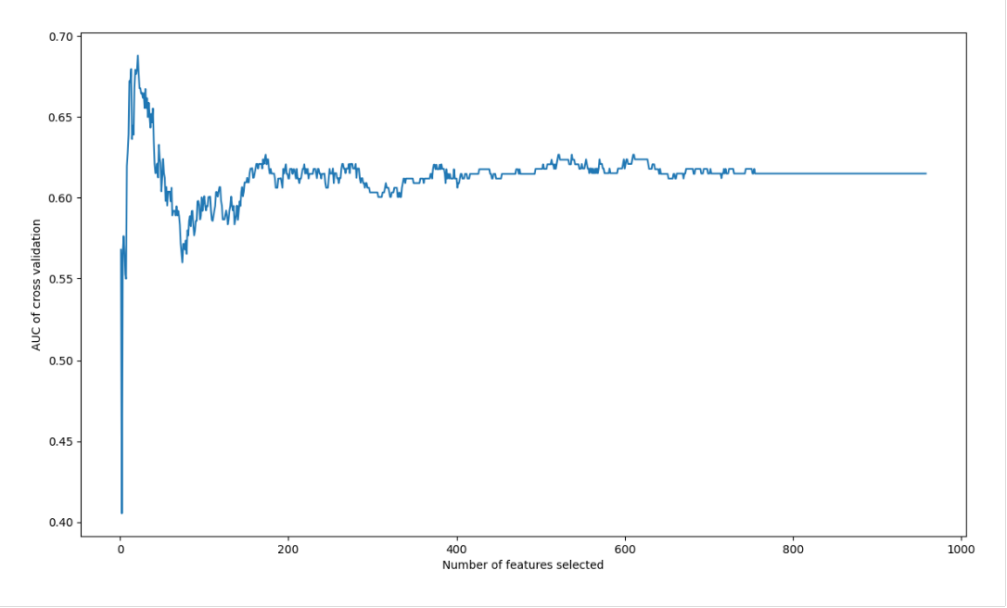
**

**Supplemental figure 2.** A five-fold cross-validation is used in the feature selection process and is calculated to evaluate them by AUC.

**
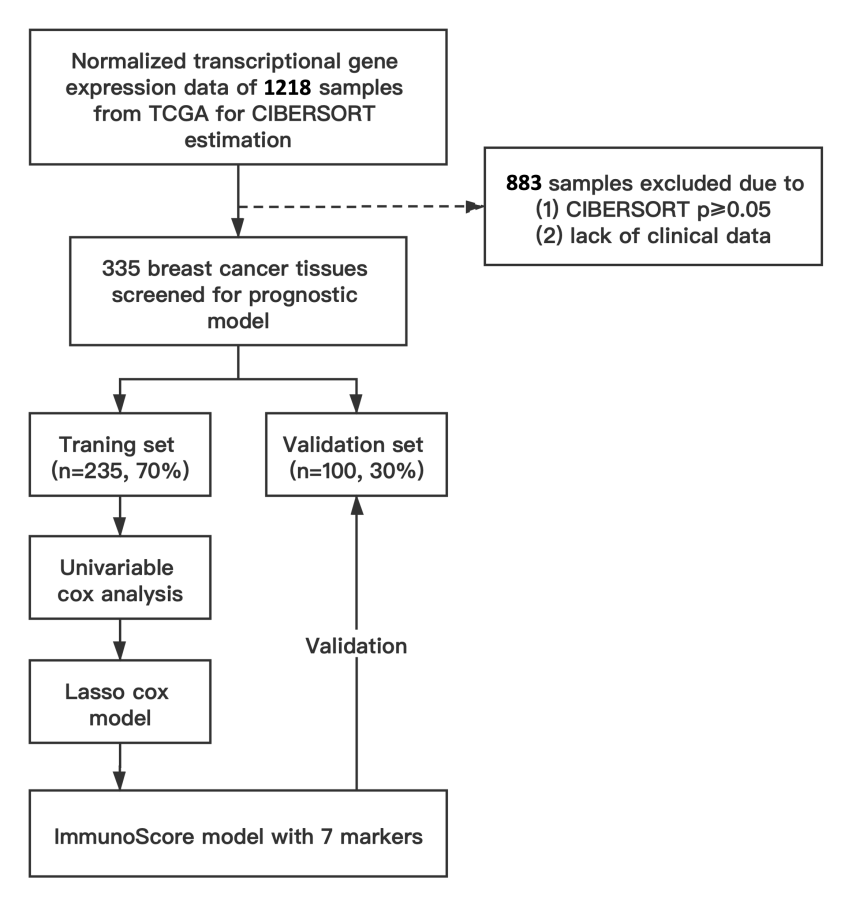
**

**Supplemental figure 3.** Process diagram showing data collection and analyses.

**
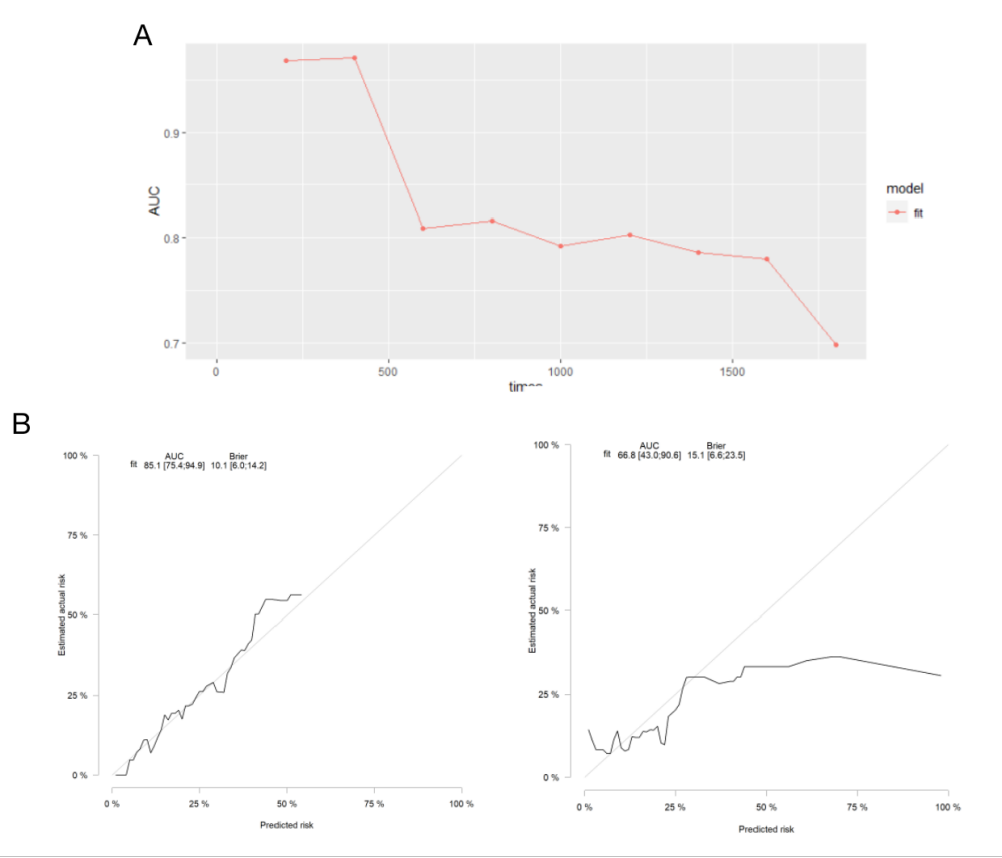
**

**Supplemental figure 4. (A)** AUC values corresponding to the time-dependent ROC analysis in the training set. **(B)** Calibration curves for the ROC curves in the training and validation sets.


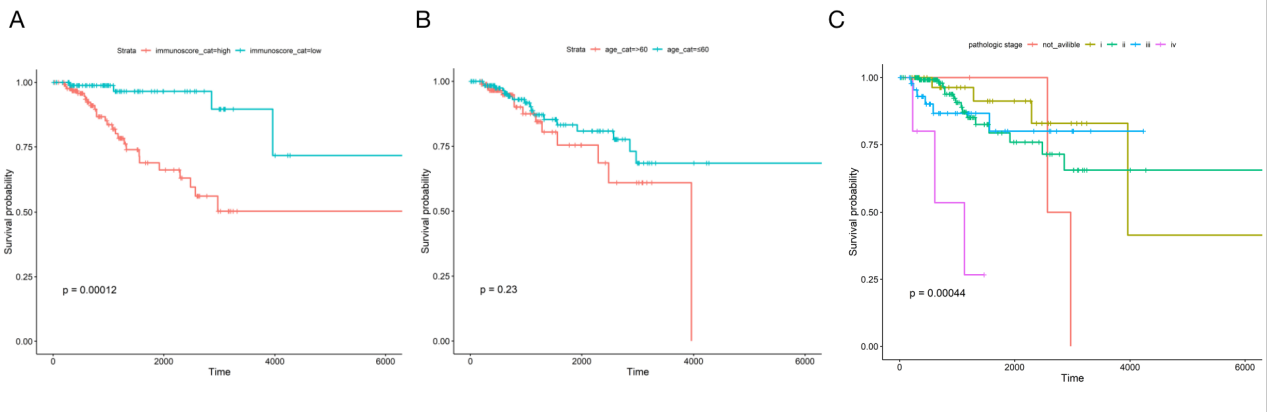
 **Supplemental figure 5.** Five-year survival analysis results for different immunoscore groups **(A)**, different age groups **(B)**, and different pathologic stages **(C)**. P-values were calculated using the log-rank test.

**
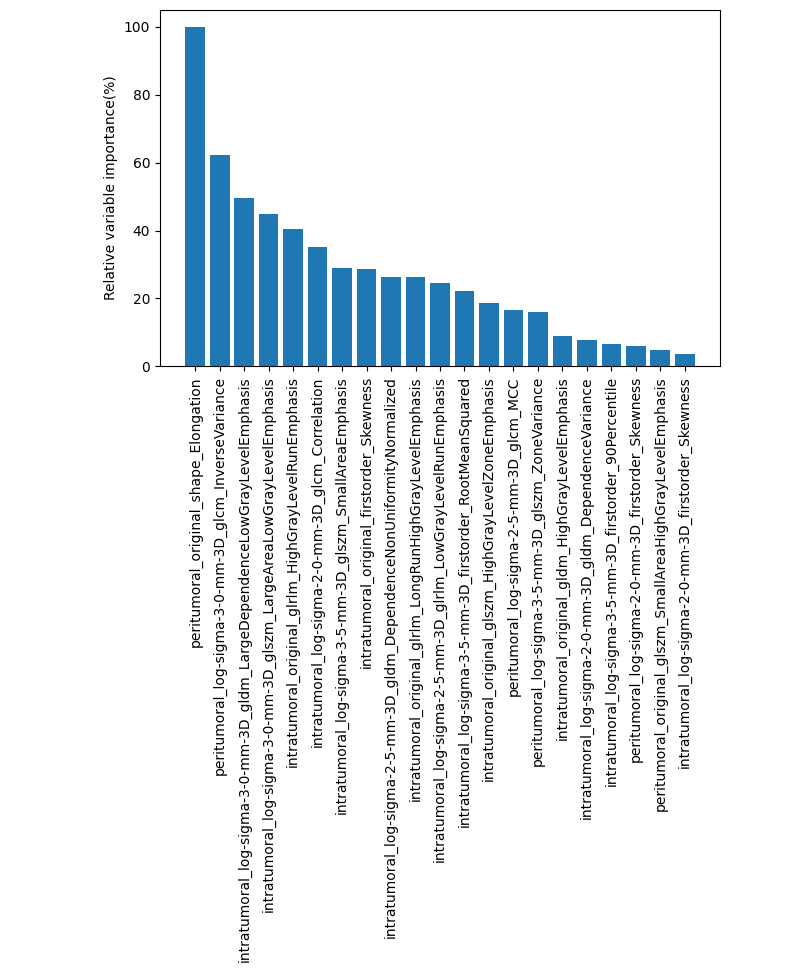
**

**Supplemental figure 6.** Histogram of the relative importance of the features of the random forest.

**
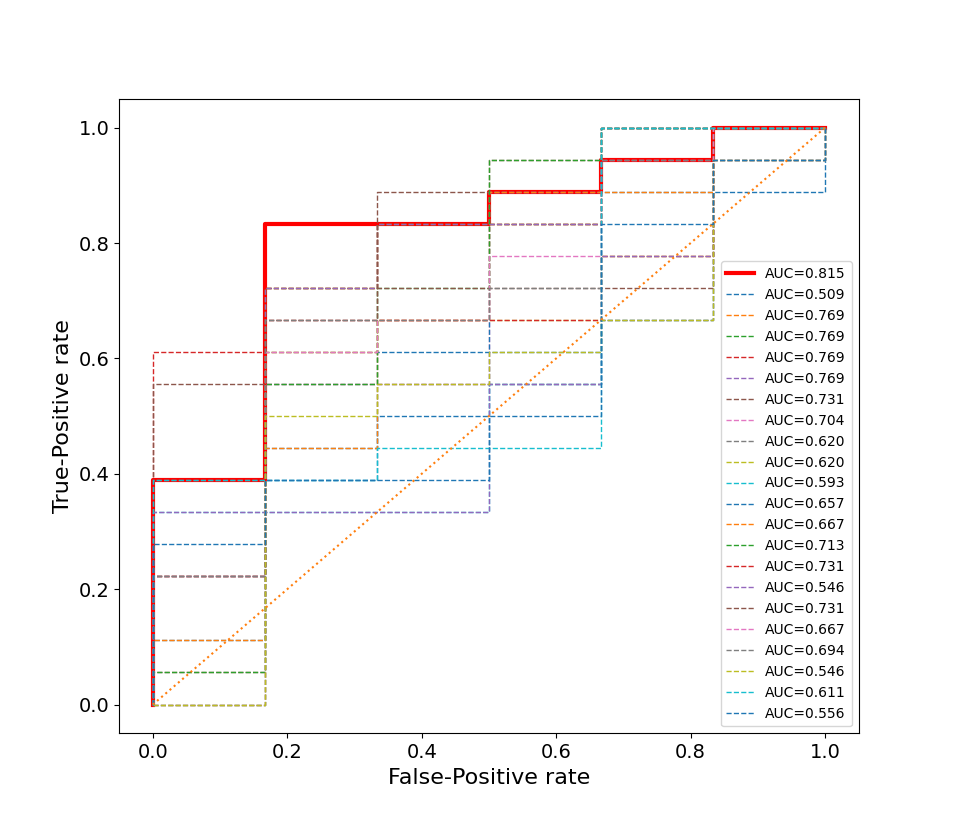
**

**Supplemental figure 7.** ROC curves for 21 chosen features (6 marginal features and 15 intratumoral features), and the RIS of the predicted IS_BC_ in the training set.

**
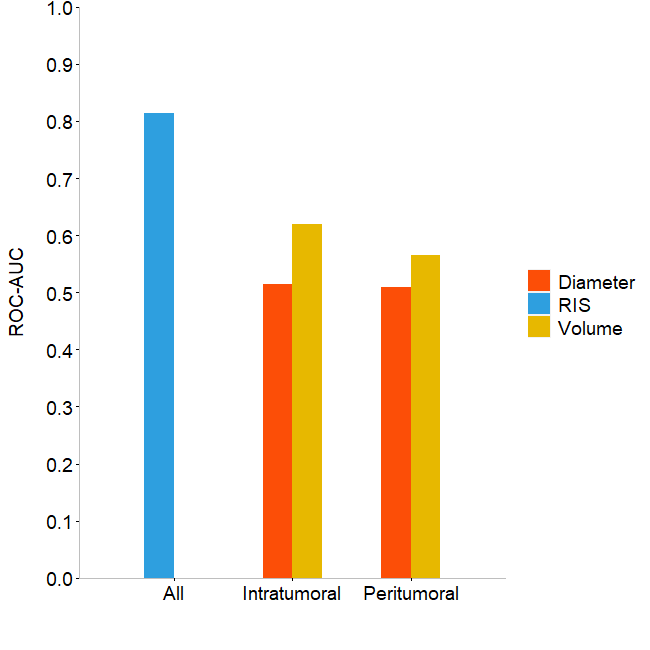
**

**Supplemental figure 8.** Comparison of the AUC of the RIS with the volume and diameter of the core, and with infiltration zones of the validation set.


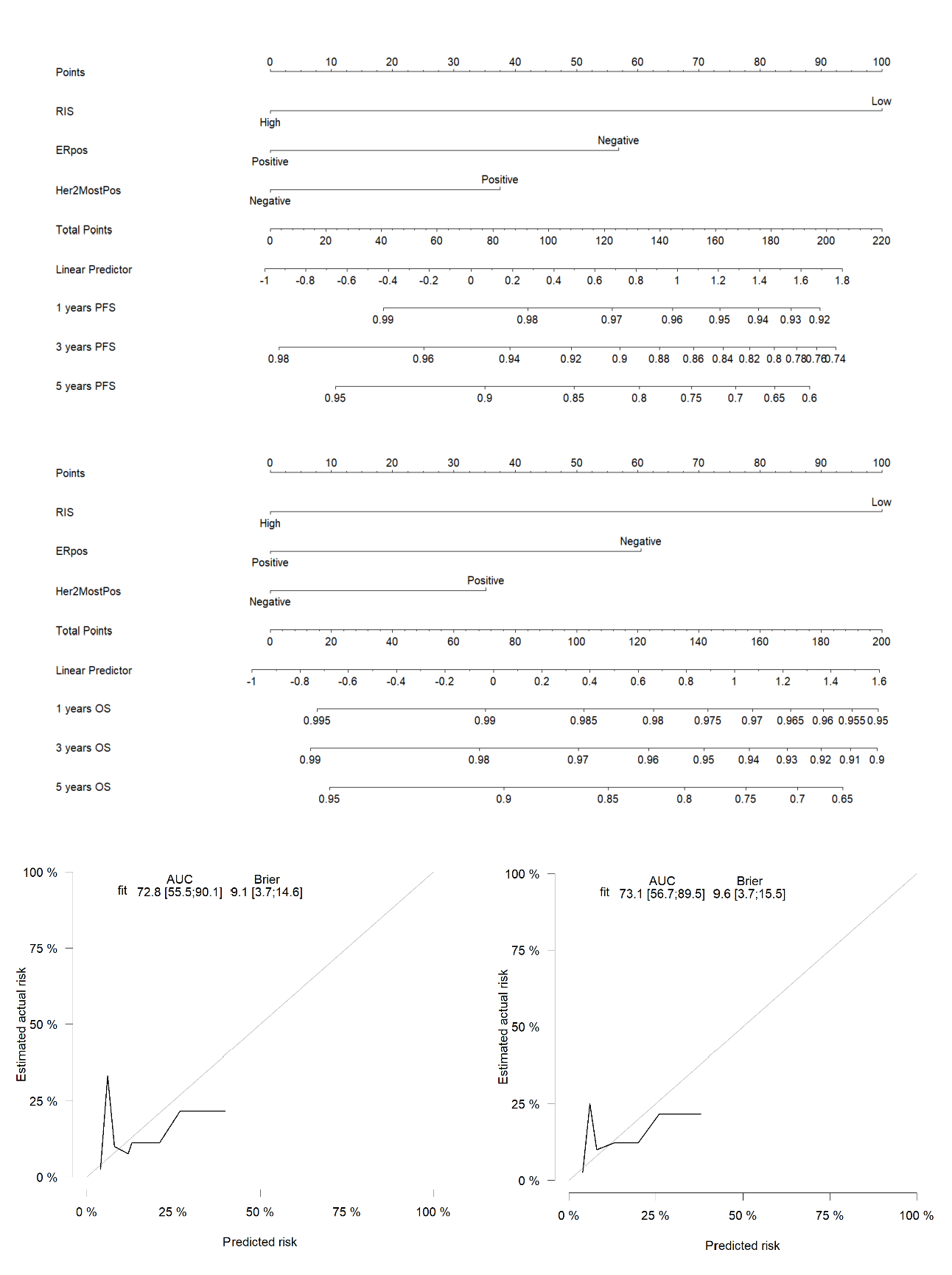


**Supplemental figure 9.** To determine the number of points a patient accepted for the RIS in terms of PFS and OS probabilities, the patient’s RIS was located in the RIS axes. A line was drawn over the point axes, and the process was repeated with every variable. The number of points obtained was summed for every risk factor, and the ultimate sum was located in the total point axes—at which point a line was drawn to identify the probability of the patient having a particular PFS and OS. The calibration curves of PFS and OS were created to evaluate the performance of the nomogram.

## Supplementary Table

| **Supplemental table 1** Results of multivariable Cox regression analysis | | | |
| --- | --- | --- | --- |
|  | Hazard ratio | 95%CI | P |
| Immunoscore-[*](https://www.ncbi.nlm.nih.gov/pmc/articles/PMC6099214/#bjs10871-note-0004) | 2.42 | 1.49—3.93 | <0.001 |
| Age-[*](https://www.ncbi.nlm.nih.gov/pmc/articles/PMC6099214/#bjs10871-note-0004) | 1.05 | 1.01—1.08 | 0.006 |
| Pathologic-stages |  |  |  |
| Ⅰ | 0.19 | 0.03, 1.08 | 0.041 |
| II | 0.51 | 0.12, 2.26 | 0.377 |
| III | 0.59 | 0.12, 2.94 | 0.516 |
| IV | 1.67 | 0.23, 12.17 | 0.610 |
| *Continuous variable. | | | |

| **Supplemental table 2.** Radiographic Feature | | | | | |
| --- | --- | --- | --- | --- | --- |
| **ROI** | **B** | **R** | **Filter** | **Class** | **Feature** |
| Intratumoral | 5 | 3.0 | Original | Firstorder | Skewness |
| Intratumoral | 5 | 3.0 | Original | GLRLM | HighGrayLevelRunEmphasis |
| Intratumoral | 5 | 3.0 | Original | GLRLM | LongRunHighGrayLevelEmphasis |
| Intratumoral | 5 | 3.0 | Original | GLSZM | HighGrayLevelZoneEmphasis |
| Intratumoral | 5 | 3.0 | Original | GLDM | HighGrayLevelEmphasis |
| Intratumoral | 5 | 3.0 | Log.sigma.2.0mm | Firstorder | Skewness |
| Intratumoral | 5 | 3.0 | Log.sigma.2.0mm | DLCM | Correlation |
| Intratumoral | 5 | 3.0 | Log.sigma.2.0mm | GLDM | DependenceVariance' |
| Intratumoral | 5 | 3.0 | Log.sigma.2.5mm | GLRLM | LowGrayLevelRunEmphasis |
| Intratumoral | 5 | 3.0 | Log.sigma.2.5mm | GLDM | DependenceNonUniformityNormalized |
| Intratumoral | 5 | 3.0 | Log.sigma.3.0mm | GLSZM | LargeAreaLowGrayLevelEmphasis |
| Intratumoral | 5 | 3.0 | Log.sigma.3.0mm | GLDM | LargeDependenceLowGrayLevelEmphasis |
| Intratumoral | 5 | 3.0 | Log.sigma.3.5mm | Firstorder | 90Percentile |
| Intratumoral | 5 | 3.0 | Log.sigma.3.5mm | GLSZM | RootMeanSquared |
| Intratumoral | 5 | 3.0 | Log.sigma.3.5mm | GLSZM | SmallAreaEmphasis |
| Peritumoral | 5 | 3.0 | Original | Shape | Elongation |
| Peritumoral | 5 | 3.0 | Original | GLSZM | SmallAreaHighGrayLevelEmphasis |
| Peritumoral | 5 | 3.0 | Log.sigma.2.0mm | Firstorder | Skewness |
| Peritumoral | 5 | 3.0 | Log.sigma.2.5mm | GLCM | MCC |
| Peritumoral | 5 | 3.0 | Log.sigma.3.0mm | GLCM | InverseVariance |
| Peritumoral | 5 | 3.0 | Log.sigma.3.5mm | GLSZM | ZoneVariance |
| ROI: Regions of Interest; B: binning; R: resampling | | | | | |

| **Supplemental table 3.** Patient clinical characteristics based on the RIS in the radiogenomic and validation cohorts. | | | | | | |
| --- | --- | --- | --- | --- | --- | --- |
| **Variables** | **Radiogenomic Cohort** | | | **validation cohort** | | |
|  | low RIS(%) | high RIS(%) | *P* | low RIS(%) | high RIS(%) | *P* |
| **Age(years),**  **median(IQR)** | 55(30-82) | 54(36-71) |  | 49(28-64) | 48(27-69) |  |
| **Age(years)** |  |  | 0.846 |  |  | 0.745 |
| ＜60 | 60(65.9%) | 16(59.3%) |  | 80(90.9%) | 48(85.7%) |  |
| ≧60 | 31(34.1%) | 11(40.7%) |  | 8(9.1%) | 8(14.3%) |  |
| **Laterality** |  |  | 0.008 |  |  | <0.001 |
| Left | 46(50.5%) | 13(48.1%) |  | 43(48.9%) | 30(53.6%) |  |
| Right | 45(49.5%) | 14(51.9%) |  | 45(51.1%) | 26(46.4%) |  |
| **Race** |  |  | 0.003 |  |  | <0.001 |
| asian | 1(1.1%) | 0(0%) |  | 4(4.6%) | 4(7.1%) |  |
| black or african american | 13(14.3%) | 8(29.6%) |  | 12(13.6%) | 15(26.8%) |  |
| white | 77(84.6%) | 19(70.4%) |  | 72(81.8%) | 37(66.1%) |  |
| **Stage** |  |  | 0.001 |  |  | / |
| I | 26(28.6%) | 1(3.7%) |  | / | / |  |
| II | 53(58.2%) | 22(81.5%) |  | / | / |  |
| III | 12(18.2%) | 4(14.8%) |  | / | / |  |
| IV | / | / |  | / | / |  |
| **ERpos** |  |  | 0.005 |  |  | <0.001 |
| positive | 76(83.5%) | 6(22.2%) |  | 56(63.6%) | 29(51.8%) |  |
| negative | 15(16.5%) | 21(77.8%) |  | 32(36.4%) | 25(44.6%) |  |
| NA | / | / |  | / | 2（3.6%） |  |
| **PgRpos** |  |  | 0.002 |  |  | <0.001 |
| positive | 69(75.8%) | 17(63.0%) |  | 48(54.5%) | 22(39.3%) |  |
| negative | 22(24.2%) | 10(37.0%) |  | 40(45.5%) | 32(57.1%) |  |
| NA | / | / |  | / | 2(3.6%) |  |
| **Her2MostPos** |  |  | 0.001 |  |  | <0.001 |
| positive | 11(12.1%) | 2(7.4%) |  | 29(33.0%) | 16(28.6%) |  |
| negative | 52(57.1%) | 10(37.0%) |  | 59(67.0%) | 37(66.1%) |  |
| NA | 28(30.8%) | 15(55.6%) |  | / | 3(5.3%) |  |

| **Supplemental table 4**. Univariate associations among RIS, clinicopathologic characteristics and progression-free and overall survival in the validation cohort. | | | | | |
| --- | --- | --- | --- | --- | --- |
| **Variables** | **Progression-free survival** | |  | **Overall survival** | |
|  | 95%CI) | *p* |  | 95%CI | *p* |
| **RIS** | **0.079-0.870** | **0.028** |  | **0.083-0.92** | **0.036** |
| Age(years) (≥60 vs. <60) | 0.429-5.126 | 0.533 |  | 0.446-5.322 | 0.495 |
| Laterality | 0.446-5.322 | 0.495 |  | 0.389-2.470 | 0.967 |
| ERpos | 0.564-1.088 | 0.145 |  | 0.542-1.046 | 0.09 |
| PgRpos | 0.607-1.162 | 0.293 |  | 0.610-1.168 | 0.306 |
| Her2MostPos | 0.572-3.835 | 0.419 |  | 0.547-3.655 | 0.474 |

| **Supplemental table 5.** Improvements in net reclassification by comparison of nomogram and RIS. | | | | | |
| --- | --- | --- | --- | --- | --- |
| Patients | **Progression-free survival** | |  | **Overall survival** | |
|  | NRI (95% CI) | *p* |  | NRI (95% CI) | *p* |
| Nomogram *vs.* RIS | |  |  |  |  |
| **Validation cohort** | 0.109 (0.024-0.477) | <0.05 |  | 0.334 (0.090-0.451) | <0.05 |
| NRI: Net reclassification improvement. | | | | | |
